# Gelatin versus its two major degradation products, prolyl‐hydroxyproline and glycine, as supportive therapy in experimental colitis in mice

**DOI:** 10.1002/fsn3.639

**Published:** 2018-04-16

**Authors:** Suqin Zhu, Min Huang, Guangxin Feng, Yu Miao, Haohao Wu, Mingyong Zeng, Yangming Martin Lo

**Affiliations:** ^1^ College of Food Science and Engineering Ocean University of China Qingdao Shandong Province China; ^2^ Department of Clinical Laboratory The Affiliated Hospital of Qingdao University Qingdao Shandong Province China; ^3^ College of Biological Science and Engineering Fuzhou University Fujian China

**Keywords:** Collagen, dextran sodium sulfate‐induced colitis, Gelatin, glycine, inflammatory bowel disease, prolyl‐hydroxyproline (Pro‐Hyp)

## Abstract

Gelatin is an anti‐inflammatory dietary component, and its predominant metabolites entering circulation are prolyl‐hydroxyproline (Pro‐Hyp) and glycine. We evaluated the protective effects of orally administered gelatin, glycine, and Pro‐Hyp 10:3:0.8 (w/w/w) against dextran sodium sulfate (DSS)‐induced colitis in mice. According to clinical, histological, and biochemical parameters, they exhibited significant activities in the order of gelatin < glycine < Pro‐Hyp. Gelatin prevented the DSS‐induced increase in interleukin‐1β (IL‐1β), interleukin‐6 (IL‐6), and tumor necrosis factor‐α (TNF‐α) in the colon, rather than in peripheral blood. Glycine and Pro‐Hyp attenuated the DSS‐induced rise in colonic IL‐6 and TNF‐α, as well as peripheral IL‐1β, IL‐6, and TNF‐α. Hematologic results show the attenuation of DSS‐induced leukocytosis and lymphocytosis by glycine and Pro‐Hyp, rather than gelatin. These findings suggest that glycine and Pro‐Hyp constitute the material basis for gelatin's anticolitis efficacy, and they have better anticolitis activities and distinct mechanisms of action when ingested as free compounds than as part of gelatin.

## 1 INTRODUCTION

Ulcerative colitis and Crohn's disease are inflammatory bowel diseases (IBD) featured by chronic intestinal inflammation. IBD affect up to 0.5% of the general population in the western world, and have become increasingly prevalent in newly industrialized countries (Kaplan, 2015). IBD are relapsing, incurable diseases leading to symptoms such as abdominal pain, persistent diarrhea, fatigue, and loss of appetite, and constitute the primary risk factor for the development of colorectal malignancy (Herszényi, Barabás, Miheller, & Tulassay, 2015). They significantly impair the patient's health‐related quality of life, and cause a considerable economic burden across the patient's lifespan (Floyd, Langham, Severac, & Levesque, 2015; Moradkhani, Beckman, & Tabibian, 2013). The pathogenesis of IBD involves an individual's genetic predisposition, environmental triggers, and abnormal immune responses (Zhang & Li, 2014). The western dietary pattern, rich in red and processed meat, high‐fat dairy products, refined grains, and high‐sugar drinks but deficient in fruits, vegetables, fish, and legumes, has been proposed as a vital environmental trigger for IBD in the western world and newly industrialized countries (Haskey & Gibson, 2017).

Long‐term pharmacotherapy for IBD has severe side effects such as drug hypersensitivity, nausea, nephrotoxicity and rash, and also encounters the problem of low medication adherence (Bhasin, Singh, Targownik, Israeli, & Bernstein, 2016; Hou, Lee, & Lewisk, 2014). A large majority (up to 71%) of IBD patients believe that diet affects their disease, and they have a strong tendency to seek dietary strategies to control or minimize symptoms (Holt, Strauss, & Moore, 2017). However, a limited number of specific dietary factors have been examined for being detrimental or protective against IBD in clinical trials or experimental models (Hou et al., 2014). This knowledge deficit hinders the development of a rich variety of nutritionally balanced anti‐IBD diets for patients, which retards the implementation of dietary strategies and approaches into practice (Richman & Rhodes, 2013).

Anti‐IBD dietary factors reported so far can be mainly categorized into polyphenols, dietary fiber, and omega‐3 polyunsaturated fatty acids (Martin & Bolling, 2015; Michalak, Mosinska, & Fichna, 2016; Rose, Demeo, Keshavarzian, & Hamaker, 2007), with only a few protein‐derived anti‐IBD factors (e.g., the peptides from soy, corn gluten and egg white) being discovered yet (Lee et al., 2009; Mochizuki, Shigemura, & Hasegawa, 2010; Young, Ibuki, Nakamori, Fan, & Mine, 2012). Collagen is the most abundant animal protein, and accounts for about 30% of total protein in both vertebrates and invertebrates (Liu, Nikoo, Boran, Zhou, & Regenstein, 2015). Collagen peptides have been reported to be anti‐inflammatory against osteoarthritis, burn injury, skin inflammation, and ulcerative colitis (Azuma, Osaki, Itoh, Arifuku, & Okamoto, 2014; Chen, Hou, Wang, Zhao, & Li, 2017; Dar et al., 2017; Hartog, Cozijnsen, de Vrij, & Garssen, 2013). However, collagen‐rich animal tissues such as skin, tendon, and cartilage are largely excluded from the modern diet, which typically includes a great deal of processed meat. Therefore, dietary inclusion of gelatin, the denatured collagen, might be beneficial to IBD patients in industrialized countries.

Glycine and prolyl‐hydroxyproline (Pro‐Hyp) are the most abundant gelatin‐derived amino acid and peptide, respectively, in the circulation (Hartog et al., 2013; Ichikawa et al., 2010), so might be the material basis for gelatin's anti‐inflammatory activity. In fact, glycine has been recognized as an anti‐inflammatory agent with a wide spectrum of protective properties including that of anticolitis (Tsune et al., 2003; Zhong et al., 2003); nevertheless, Pro‐Hyp has so far not been examined for its anti‐inflammatory activity. This study aimed to clarify the efficacy and mechanism of action of dietary gelatin as ancillary treatment in IBD. We evaluated the protective effects of orally administered gelatin, glycine, and Pro‐Hyp, in mice with dextran sodium sulfate (DSS)‐induced colitis, followed by an investigation of the mechanism of action based on tissue and serum cytokines and hematologic parameters.

## 2 MATERIALS AND METHODS

### 2.1 Reagents

Gelatin from porcine skin, glycine, o‐dianisidine dihydrochloride, phenylmethanesulfonyl fluoride (PMSF), and the total glutathione (tGSH, reduced plus oxidized forms) assay kit were provided by Sigma‐Aldrich Co. (Shanghai, China). DSS (molecular weight, 36–50 kDa) was purchased from Yisheng Biotechnology Co., Ltd. (Shanghai, China). The adenosine 5′‐triphosphate (ATP) determination kit, the Pierce BCA protein assay kit, and sandwich enzyme‐linked immunosorbent assay (ELISA) kits for mouse tumor necrosis factor‐α (TNF‐α), interleukin‐1β (IL‐1β), and interleukin‐6 (IL‐6) were from ThermoFisher Scientific (San Jose, CA, USA). Pro‐Hyp was provided by GL Biochem Ltd. (Shanghai, China) at purities >98%. Radioimmunoprecipitation assay (RIPA) lysis buffer [50 mmol/L Tris‐HCl pH 7.4, 150 mmol/L NaCl,1% Triton X‐100,1% sodium deoxycholate,and 0.1% SDS] and protease and phosphatase inhibitor cocktails were obtained from Beyotime Biotechnology (Shanghai, China). Other reagents used were of analytical grade and commercially available.

### 2.2 Animal experiments

Male C57BL/6 mice (7–8 weeks old, weighing 22 ± 1.83 g) were purchased from Lukang Pharmaceutical Co. (Shandong, China). Animals were housed in individual cages in an air‐conditioned room (20–24°C, 55%–65% humidity) with a lighting cycle of 12‐h light and 12‐h dark, and received a standard laboratory chow diet (TROPHIC Animal Feed High‐Tech Co., Ltd., Nantong, China) *ad libitum* throughout the experiment. All experiments were carried out ethically according to the principles in the National Institutes of Health (NIH) *Guide for the Care and Use of Laboratory Animals* and were approved by the Committee on the Ethics of Animal Experiments of Ocean University of China.

**Figure 1:**
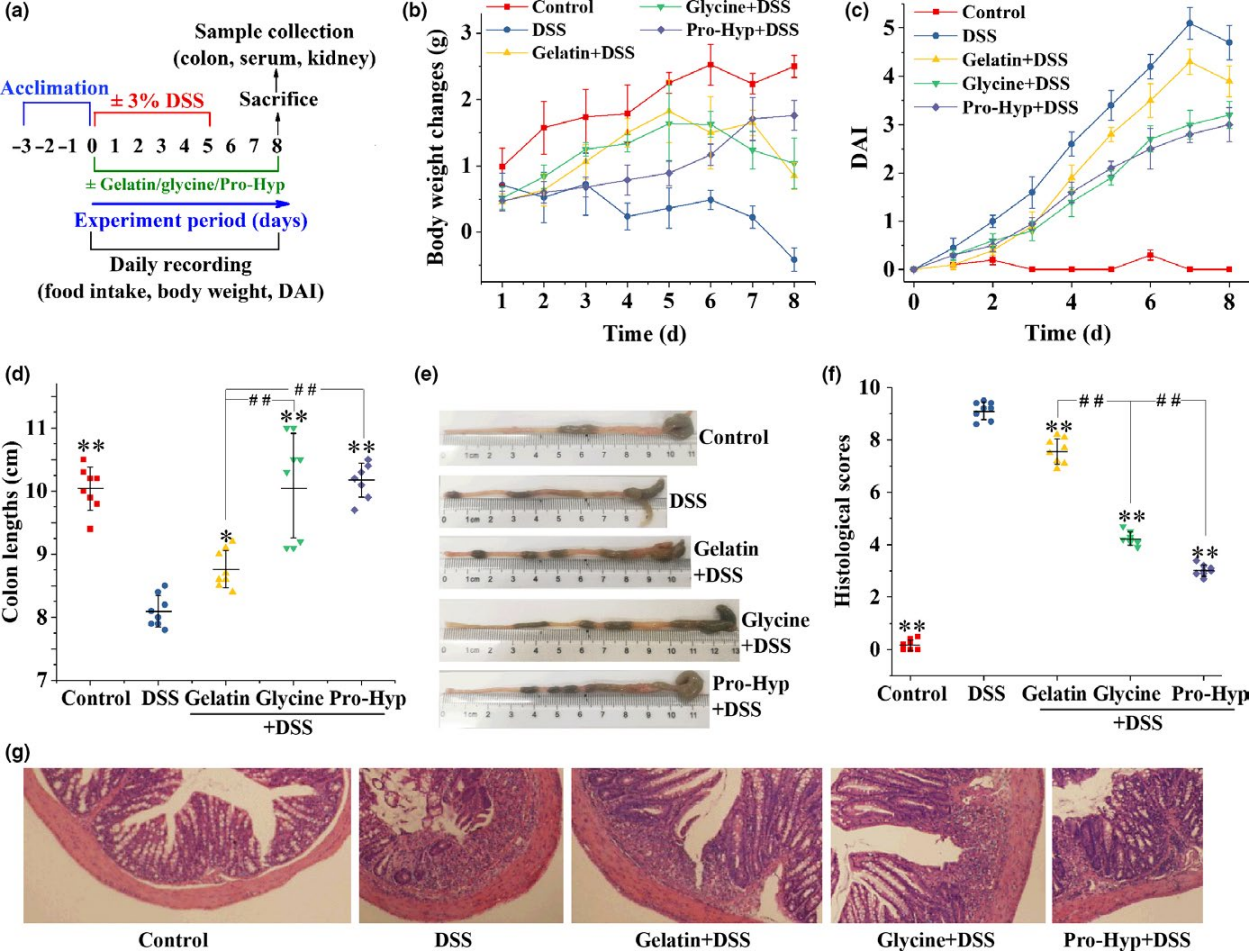
Clinical and histological parameters of DSS‐induced colitis: (a) the experimental plan; (b) body weight changes, represented as means ± SD, compared to initial body weight of each group; (c) disease activity index (DAI), represented as means ± SD; (d) statistical analysis (e) representative images of colon length; (f) histological scores, and (g) representative H&E images (original magnification, 400 × ) of the colon tissue. Each point in (d) and (f) represents a single sample, with significance marked as ** *p *<* *.01 and * *p *<* *.05 compared to the DSS group, or ## *p *<* *.01 compared between groups

Following three days of acclimation, the mice were randomly assigned to five experimental groups (*n *= 8 per group): (1) the control group, which received Milli‐Q water *ad libitum*, and daily intragastric administration of 200 μl Milli‐Q water for an 8‐d experimental period; (2) the DSS group, which received 3% (w/v) DSS *ad libitum* for 5 days before changing to Milli‐Q water for another 3 days, and daily intragastric administration of 200 μl Milli‐Q water for the course of the entire experiment; (3) the test groups, which received DSS as described above, and daily intragastric administration of 200 μl of 30% (w/v) gelatin, 9% (w/v) glycine, or 2.5% (w/v) Pro‐Hyp for the course of the entire experiment. The experimental plan is shown in Figure 1a. The DSS solution was changed everyday. Thirty g of gelatin was dissolved in 70 ml Milli‐Q water by warming at 60°C, and then the solution was diluted to 100 ml with water to obtain the 30% (w/v) gelatin solution. The solutions of gelatin, glycine, and Pro‐Hyp were warmed to 37°C before being administered, and no gelling was observed in the gelatin solution. The dosage ratio of gelatin, glycine, and Pro‐Hyp at 10:3:0.8 (w/w/w) was corresponding to the approximate proportions of glycine and Pro‐Hyp in collagen.

Food intake was recorded everyday. Mice were weighed daily and inspected for clinical symptoms including stool consistency and fecal blood (Laroui et al., 2012). Stool consistency scoring is defined as follows: 0 = normal; 1 = moist/sticky stool; 2 = soft stool; 3 =  diarrhea. Fecal blood scoring is defined as follows: 0 =  no blood; 1 =  evidence of blood in stool or around anus; 2 =  severe bleeding. Disease activity index (DAI) was the sum of the scores of stool consistency and fecal blood. Colon and blood were sampled at the end of the experiment on day 8. The length of the entire colon from the ileocecal junction to the anus was measured at rest, without stretching. The proximal colon was cut into small pieces (~1 cm long) for histological and biochemical analysis. Whole blood samples were left to clot for about 30 min at room temperature, before serum was obtained by centrifugation at 3,000 rpm for 15 min at 4°C. The blood samples for hematological analysis were collected in tubes containing ethylenediamine tetra‐acetic acid (EDTA). Hemoglobin, white blood cells, and lymphocytes in the blood samples containing EDTA were analyzed on a XN‐9000 hematology analyzer (Sysmex, Kobe, Japan), and all hemolyzed or clotted samples were discarded before analysis.

### 2.3 Histopathology

For routine hematoxylin and eosin (H&E) staining, proximal colon tissues were fixed in 10% neutral‐buffered formaldehyde and desiccated and embedded in paraffin. Sections (thickness, 5 μm) made from the paraffin‐embedded tissues were stained with H&E for histologic observation. Six randomly chosen fields for each sample were photographed using a OLYMPUS DP70 microcamera and the DP controller software (version 3.2.1.276) built in a OLYMPUS BX41 microscope (Japan). The histologic lesion in each field was scored as described by Laroui et al., 2012. A mean score for six fields was used as the histologic score for each mouse.

### 2.4 Myeloperoxidase (MPO) assay

MPO activities in proximal colon tissues were measured as previously reported (Rodriguez‐Palacios, Aladyshkina, & Cominelli, 2015). Preweighed tissue samples (stored at −80°C for no more than 48 h) were homogenized in 20 volumes of ice‐cold potassium phosphate buffer (50 mmol/L K_2_HPO_4_ and 50 mmol/L KH_2_PO_4_, pH 6.0) containing 0.5% hexadecyltrimethylammonium bromide using the Bioprep‐6 Homogenizer (Aosheng, China) in the presence of ceramic beads. The homogenates were centrifuged at 12,500 rpm for 5 min at 4°C. After loading of 10 μl sample supernatant to each well on the prechilled 96‐well plates, 200 μl of active dianisidine substrate containing 0.2 mg/ml o‐dianisidine hydrochloride and 0.001% H_2_O_2_ was added to each sample well, and the absorbance at 450 nm was measured in a Synergy H4 hybrid microplate reader (Bio‐Tek, Winooski, VT, USA) every 30 s for 5 min at 28°C. One unit of MPO activity was calculated as: MPO activity = ΔA450 ÷ 0.5 ÷ 0.0113 ÷ 0.05; where ΔA450 was the average of ΔA450_(t30‐t0)_ (the absorbance difference from time 0 s to 30 s) and ΔA450_(t60‐t30)_ (the absorbance difference from time 30 s to 60 s); 0.0113 is MPO constant; 0.5 is for time intervals (0.5 min), and 0.05 is dilution factor of sample:HTBA lysis buffer (50 mg:1 ml).

### 2.5 Quantification of tGSH in the tissue

Proximal colon tissues were homogenized in 10 volumes of the 5% 5‐sulfosalicylic acid solution using the Bioprep‐6 Homogenizer (Aosheng, China) in the presence of ceramic beads. The homogenate was centrifuged at 10,000 rpm for 5 min at 4°C, and the supernatant was used as the tissue extract. The level of tGSH in the tissue extract was determined using the tGSH assay kit according to the manufacturer's instructions.

### 2.6 ATP determination assay

Phenol–TE reagent was prepared as described previously (Chida & Kido, 2014). Proximal colon tissues (30–70 mg) were homogenized in 1 ml of ice‐cold Phenol–TE reagent using the Bioprep‐6 Homogenizer (Aosheng, China) in the presence of ceramic beads. The homogenate was centrifuged at 10,000 rpm for 5 min at 4°C, and the supernatant was used as the tissue extract. The level of ATP in the tissue extract was determined using the ATP determination assay kit according to the manufacturer's instructions.

### 2.7 Measurements of tissue and serum levels of cytokines

Proximal colon tissues were homogenized in 25 volumes of RIPA lysis buffer supplemented with 1 mmol/L PMSF and protease and phosphatase inhibitor cocktails using the Bioprep‐6 Homogenizer (Aosheng, China) in the presence of ceramic beads. The homogenate was centrifuged at 10,000 rpm for 5 min at 4°C, and the supernatant was used as the tissue extract. Protein concentration in the tissue extract was measured with Pierce BCA protein assay kit. Levels of IL‐1β, IL‐6, and TNF‐α in serum and the tissue extract were determined using ELISA kits according to the manufacturer's instructions.

### 2.8 Statistical analysis

Statistical analyses were performed using SPSS software version 19.0 (SPSS, Inc., Chicago, USA) and OriginPro 7.0 software (OriginLab Co., Northampton, USA). Data were expressed as means ± standard deviations (SD). The mean differences were compared by one‐way ANOVA with Tukey's HSD test. All the differences were considered to be significant and very significant at *p *<* *.05 and *p *<* *.01, respectively.

## 3 RESULTS

### 3.1 Clinical and histological parameters of colitis

Some clinical signs of colitis were measured to investigate the anticolitis effects of oral gelatin, glycine, and Pro‐Hyp. The DSS‐treated mice had a significantly lower average total food intake (23.3 ± 1.1 g/mouse) during the entire experiment of 8 days than the vehicle controls (28.1 ± 2.0 g/mouse) (*p *<* *.01), while the gelatin, glycine, and Pro‐Hyp groups gave average total food intakes of 31.1 ± 3.3 g/mouse, 31.4 ± 2.4 g/mouse, and 31.1 ± 2.9 g/mouse, respectively, showing no significant differences with the control group. As shown in Figure 1b, the DSS treatment induced significant body weight loss compared to the vehicle controls from day 2 (*p *<* *.01), while from day 4, the gelatin, glycine, and Pro‐Hyp groups had significantly less body weight loss than did the DSS group (*p *<* *.01). The DSS exposure caused loose stool and fecal blood, as estimated with the DAI values (Figure 1c), and this was ameliorated by the supplementations of gelatin, glycine, and Pro‐Hyp from day 2 (*p *<* *.01), with a remarkably weaker protecting effect observed for gelatin from day 5 (*p *<* *.01). The DSS‐induced shortening of the colon was also significantly improved by the supplementations of gelatin (*p *<* *.05), glycine (*p *<* *.01), and Pro‐Hyp (*p *<* *.01), with gelatin showing a less extent of amendment compared to glycine (*p *<* *.01) and Pro‐Hyp (*p *<* *.01) (Figure 1d,e).

Colon sections stained with H&E were used to directly examine colonic inflammation (Figure 1g). DSS‐treated mice displayed thinned and disordered mucosal structure, and massive infiltration of neutrophil and lymphocyte into the mucosa and submucosa. The supplementations of gelatin, glycine, and Pro‐Hyp clearly ameliorated the abnormality of mucosal structure, and reduced the area of inflammatory cell infiltration. As shown in Figure 1f, the DSS group displayed significant greater histological scores than the control group (*p *<* *.01), while the gelatin, glycine, and Pro‐Hyp groups had significantly lower histological scores than the DSS group (*p *<* *.01), with the protecting activities following the order gelatin < glycine < Pro‐Hyp (*p *<* *.01).

### 3.2 Biochemical parameters

**Figure 2:**
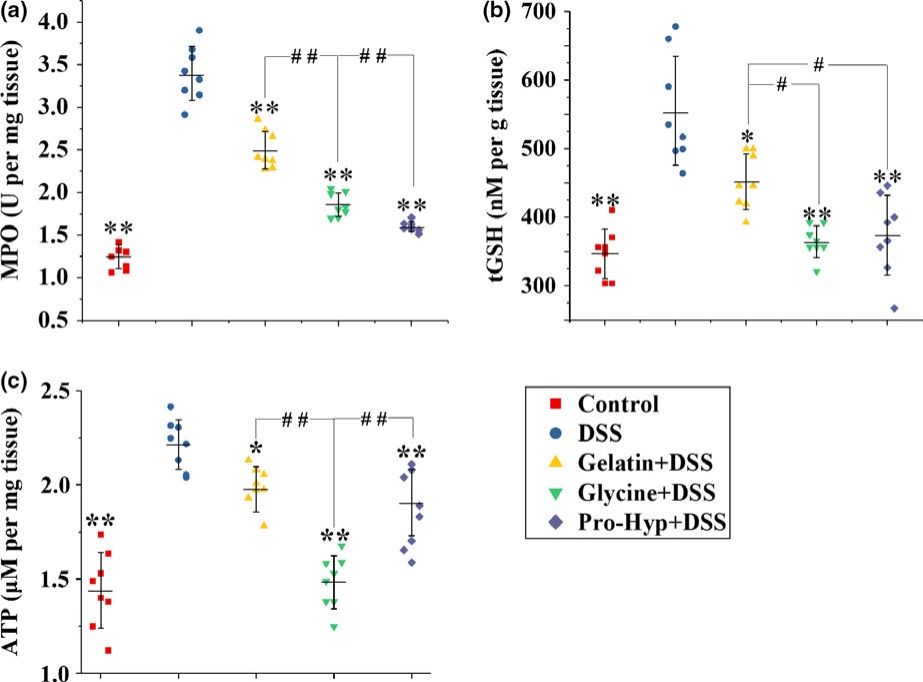
Determination of (a) MPO activity, (b) tGSH level, and (c) ATP content in colon tissues from mice in different groups. Each point represents a single sample, with significance marked as ** *p *<* *.01 and *p <* *.05 compared to the DSS group, or ##*p *<* *.01 and #*p *<* *.05 compared between groups

The activity of MPO, the most abundant protein in neutrophils, is widely used as an index of inflammation. The DSS treatment lead to significantly higher MPO activity in the colon tissue (*p *<* *.01), while the supplementations of gelatin, glycine, and Pro‐Hyp effectively attenuated the DSS‐induced rise in colonic MPO activity (*p *<* *.01), with the protecting activities following the ascending order gelatin, glycine, and Pro‐Hyp (*p *<* *.01) (Figure 2a). In many chronic inflammatory diseases, GSH consumption rate can increase dramatically to protect tissues from reactive oxygen species (ROS) liberated by recruited phagocytes (Moura, de Andrade, dos Santos, Araujo, & Goulart, 2015). Compared to the control group, the DSS group showed a significantly higher level of colonic tGSH (*p *<* *.01) (Figure 2b). This DSS‐induced rise in colonic tGSH level was significantly inhibited by the supplementations of gelatin (*p *<* *.05), glycine (*p *<* *.01), and Pro‐Hyp (*p *<* *.01), with gelatin showing a significantly lower activity than glycine and Pro‐Hyp (*p *<* *.05). The colonic ATP level can rise during inflammation due to the release of intracellular ATP from injured cells (Matsukawa et al., 2016). In this study, DSS treatment caused a significant augmentation in colonic ATP level (*p *<* *.01), and this effect was effectively attenuated by the supplementations of gelatin (*p *<* *.05), glycine (*p *<* *.01), and Pro‐Hyp (*p *<* *.01), with glycine showing a significantly higher activity than gelatin and Pro‐Hyp (*p *<* *.01) (Figure 2c).

### 3.3 Cytokine levels in the colonic tissue and serum

**Figure 3:**
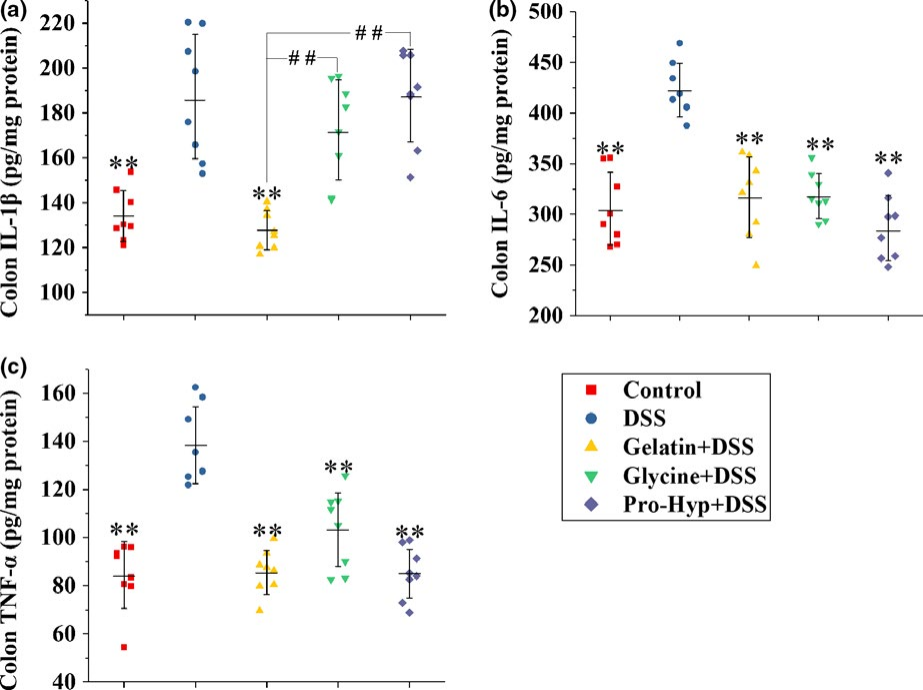
Levels of (a) IL‐1β, (b) IL‐6 and (c) TNF‐α in colon tissues from mice in different groups. Each point represents a single sample, with significance marked as **p <* *.01 compared to the DSS group, or ##*p *<* *.01 compared between groups

**Figure 4:**
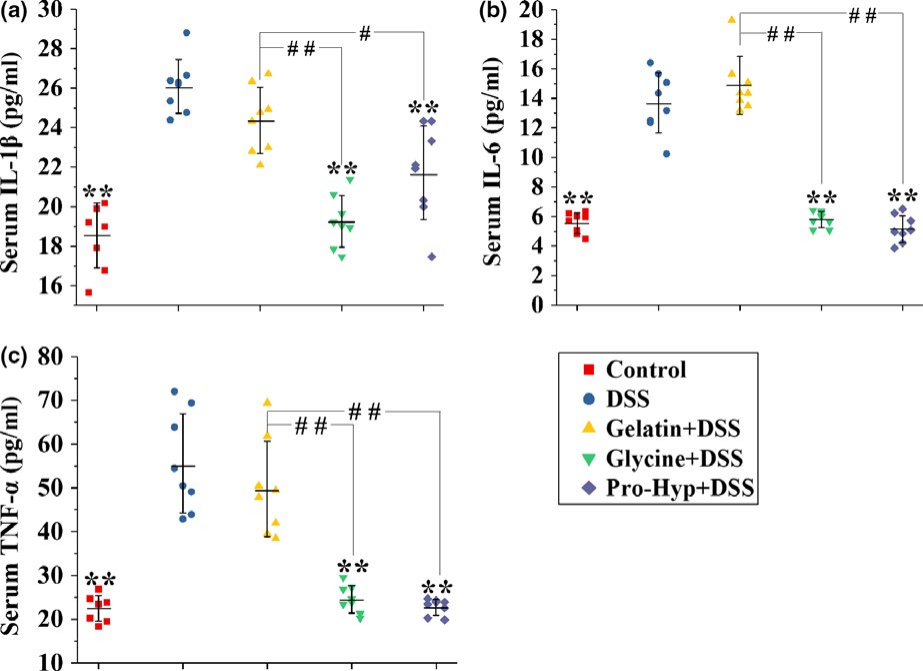
Levels of (a) IL‐1β, (b) IL‐6 and (c) TNF‐α in serum from mice in different groups. Each point represents a single sample, with significance marked as **p <* *.01 compared to the DSS group, or ##*p *<* *.01 and #*p *<* *.05 compared between groups

Figures 3 and 4 show the levels of cytokines in the colonic tissue and serum, respectively. The DSS treatment significantly elevated IL‐1β, IL‐6, and TNF‐α levels in the colon and serum (*p *<* *.01). Gelatin supplementation attenuated the elevation of colonic cytokine levels (*p *<* *.01), but exerted no effect on serum cytokines. In contrast, the supplementations of glycine and Pro‐Hyp inhibited the increase in all three cytokines in serum (*p *<* *.01), but had no effect on colonic IL‐1β. The supplementations of glycine and Pro‐Hyp also prevented the DSS‐induced rise in colonic IL‐6 and TNF‐α (*p *<* *.01).

### 3.4 Hematologic results

**Figure 5:**
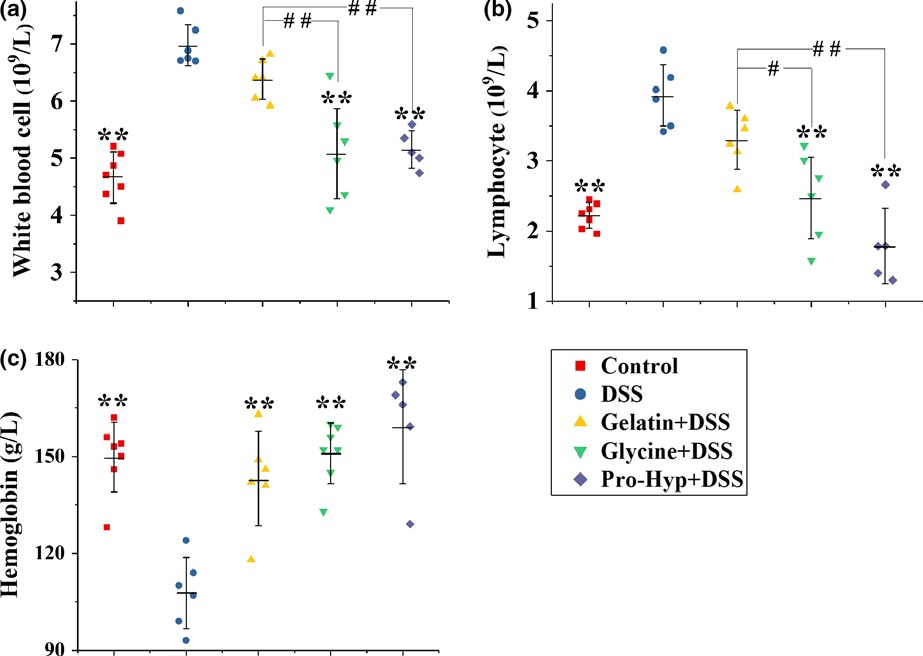
The hematologic results of mice in different groups: (a) white blood cells, (b) lymphocytes, and (c) hemoglobin. Each point represents a single sample, with significance marked as **p <* *.01 compared to the DSS group, or ##*p *<* *.01 and #*p *<* *.05 compared between groups

The count of white blood cells (WBC, also called leukocytes) is a widely used marker of systemic inflammation. Among WBC, lymphocytes are those involved in immunoglobulin synthesis and immune response. The DSS group displayed significantly higher levels of the counts of WBC and lymphocytes than the control group (*p *<* *.01) (Figures 5a,b), validating the DSS‐induced systemic inflammation. The supplementations of glycine and Pro‐Hyp, rather than gelatin, remarkably blunted this increase in WBC and lymphocytes (*p *<* *.01). Anemia is often encountered in chronic inflammatory diseases including IBD (Gasche, Lomer, Cavill, & Weiss, 2004). In this study, the DSS treatment significantly reduced the hemoglobin level (*p *<* *.01), while all three supplementations effectively prevented such a decrease (*p *<* *.01) (Figure 5c).

## 4 DISCUSSION

To elucidate whether glycine and Pro‐Hyp could explain the anti‐inflammatory activity of dietary gelatin, we administered gelatin, glycine, and Pro‐Hyp to mice with DSS‐induced colitis by gavage at a dose ratio of 10:3:0.8 (w/w/w), corresponding to the approximate proportions of glycine and Pro‐Hyp in collagen. The results of clinical signs, colon histology, and biochemical parameters uniformly revealed their remarkable anticolitis activities following the order gelatin < glycine < Pro‐Hyp. Tsune et al. (2003) reported that a diet containing 5% glycine prevented colitis induced by 2,4,6‐trinitrobenzene sulfonic acid (TNBS) and DSS in the rat. Heimesaat et al. (2012) discovered the amelioration of DSS‐induced colitis by intraperitoneal treatment with the synthetic hydroxyproline‐containing collagen analogue (Gly–Pro–Hyp)_10_. Ramadass et al. (2016) revealed that the intrarectal administration of both collagen and collagen peptides promoted mucosal healing in ulcerative colitis in mice. According to these results, both glycine and Pro‐Hyp seem to constitute the material basis for gelatin's anticolitis efficacy.

Although gelatin has a digestibility as high as 98.8% (Reuterswärd & Fabiansson, 2010), it displayed a significantly lower anticolitis activity than glycine and Pro‐Hyp according to DAI, colon lengths, colon histology, and biochemical parameters (Figures 1 and 2). This is probably because gelatin is not completely digested and absorbed in the small intestine (Chen, Rogers, & Harper, 1962; Nixon & Mawer, 1970). Dietary protein is absorbed by intestinal epithelial cells only after being digested into amino acids or di‐ and tripeptides (Nixon & Mawer, 1970). A great portion of gelatin's digestion products in the small intestine was found to be Hyp‐containing oligopeptides with an average of approximate six residues (Chen et al., 1962), and considering the high digestibility of gelatin, these oligopeptides should be further digested into amino acids or di‐ and tripeptides in the colon before being absorbed there. In fact, colonic bacteria have been shown to possess high proteolytic activities (Macfarlane, Cummings, & Allison, 1986), and amino acids and di‐ or tripeptides can be absorbed in mammalian distal colon by diffusion and the peptide transporter PEPT1, respectively (Binder, 1970; Wuensch et al., 2013). Orally administered glycine and Pro‐Hyp, which can be very efficiently absorbed in the small intestine (Craft, Geddes, Hyde, Wise, & Matthews, 1968; Yamamoto et al., 2015), should therefore induce higher peak concentrations of glycine and Pro‐Hyp in circulating blood than gelatin, and this may explain their superior anticolitis activities.

Following DSS‐induced impairment of lumen‐lining epithelial cells, luminal antigens or microorganisms can invade lamina propria and systemic circulation, where they activate mucosal inflammatory cells (MICs) and peripheral blood mononuclear cells (PBMCs), respectively, to secret proinflammatory cytokines such as IL‐1β, IL‐6, and TNF‐α (Jansky, Reymanova, & Kopecky, 2003; Meixenberger et al., 2010; Seo et al., 2015). Oral glycine and Pro‐Hyp, rather than gelatin, inhibited the DSS‐induced increase in IL‐1β, IL‐6, and TNF‐α in circulating blood (Figure 4), indicating that peripheral concentrations of glycine and Pro‐Hyp following oral ingestion of glycine and Pro‐Hyp, rather than gelatin, were high enough to attenuate the PBMC production of these proinflammatory cytokines. Peripheral proinflammatory cytokines, especially TNF‐α and IL‐6, are crucial mediators to induce the mobilization of immune cells such as monocytes and lymphocytes from bone marrow or thymus into the blood (Barna et al., 1994; Park et al., 2000; Ulich, del Castillo, & Guo, 1989). The DSS‐induced leukocytosis and lymphocytosis were attenuated by oral glycine and Pro‐Hyp, rather than gelatin (Figure 5a,b), which was in line with the results of serum cytokines. Therefore, probably via inhibiting the elevation of cytokine levels in the blood, oral glycine and Pro‐Hyp prevented an increased mobilization of immune cells into systemic circulation, which at least in part accounts for their superior anticolitis activities than gelatin.

Gelatin supplementation effectively prevented the DSS‐induced rise in colonic levels of IL‐1β, IL‐6, and TNF‐α (Figure 3), despite the fact that more infiltration of inflammatory cells occurred in the gelatin group (Figures 1f,g), so glycine and Pro‐Hyp *in situ* absorbed by epithelial cells, following the colonic digestion of Hyp‐containing gelatin oligopeptides, seem to be abundant enough to inhibit the MIC production of proinflammatory cytokines. Following epithelial injury, resident phagocytes of the colon release IL‐1β, rather than IL‐6 and TNF‐α, in response to the invasion of enteric pathogens such as *Salmonella* and *Pseudomonas* (Franchi et al., 2012). Oral glycine and Pro‐Hyp were incapable of attenuating the DSS‐induced rise in colonic IL‐1β (Figure 3a), so peripheral glycine and Pro‐Hyp seem not accessible enough to the colonic tissue for an action on resident phagocytes. As evidenced by tissue ATP levels (Figure 2c), glycine displayed a better activity in preventing colonic injury than gelatin and Pro‐Hyp, probably because beside its anti‐inflammatory activity (Zhong et al., 2003), glycine can also exert an antiapoptotic effect against tissue injuries (Jacob, Ascher, Hingorani, & Kallakuri, 2003; Lu et al., 2012; Zhong et al., 2012). IL‐6 and TNF‐α in the colonic tissue are mainly secreted by newly recruited myeloid cells during IBD (Smith, Ochsenbauer‐Jambor, & Smythies, 2005). Oral glycine and Pro‐Hyp attenuated the recruitment of inflammatory cells into the colonic tissue (Figure 1f,g), possibly owning to their prevention of leukocytosis and lymphocytosis (Figure 5a,b), and this may explain their effectiveness in suppressing the colonic increase in IL‐6 and TNF‐α (Figure 3b,c).

Anemia is a common complication of IBD, and impairs patients’ quality of life (Gasche et al., 2004). In this study, oral gelatin, glycine, and Pro‐Hyp effectively prevented the DSS‐induced anemia (Figure 5c). Circulating cytokines play vital roles in the etiology of anemia in IBD. IL‐6 induces the upregulation of hepcidin which inhibits intestinal iron absorption, while TNF‐α negatively affects erythropoiesis by suppressing the growth of erythroid progenitor cells (Matsukawa et al., 2016). Oral gelatin was ineffective in attenuating the increase in serum cytokines (Figure 4), but prevented the colitis‐associated anemia effectively. It thus seems that the antianemia activity of oral gelatin during IBD was independent on its anti‐inflammatory activity.

## 5 CONCLUSIONS

Glycine and Pro‐Hyp have higher anticolitis activities when ingested as free compounds than as part of gelatin. Gelatin's anticolitis activity possibly depends on the anti‐inflammatory effects of glycine and Pro‐Hyp *in situ* absorbed by the colon. Oral glycine and Pro‐Hyp perform their anticolitis activities probably by means of circulating glycine and Pro‐Hyp, which inhibit peripheral cytokine production thereby preventing leukocyte mobilization into the circulation. However, cellular studies using immune cells are needed to further characterize the anti‐inflammatory effects of glycine and Pro‐Hyp.

## CONFLICT OF INTEREST

There are no conflict of interests to declare.
